# Complexity and technological evolution: What everybody knows?

**DOI:** 10.1007/s10539-017-9603-1

**Published:** 2017-11-22

**Authors:** Krist Vaesen, Wybo Houkes

**Affiliations:** 1 Department Philosophy and Ethics, Eindhoven University of Technology, Postbox 513, 5600 MB Eindhoven, The Netherlands; 2 Faculty of Archaeology, Leiden University, Einsteinweg 2, 2333 CC Leiden, The Netherlands

**Keywords:** Cultural evolution, Cultural-evolutionary theory, Cumulative culture, Complexity, Technology

## Abstract

The consensus among cultural evolutionists seems to be that human cultural evolution is cumulative, which is commonly understood in the specific sense that cultural traits, especially technological traits, increase in complexity over generations. Here we argue that there is insufficient credible evidence in favor of or against this technological complexity thesis. For one thing, the few datasets that are available hardly constitute a representative sample. For another, they substantiate very specific, and usually different versions of the complexity thesis or, even worse, do not point to complexity increases. We highlight the problems our findings raise for current work in cultural-evolutionary theory, and present various suggestions for future research.

## Introduction

One aspect of contemporary culture is the rapid advance and diffusion of technologies, many of which—surgical robots, fighter planes, the latest version of your operating system—require highly specialized skills and knowledge to manufacture, maintain and operate, and have more parts than there are ants in the average anthill. As even a cursory inspection of the history of technology shows, this is hardly distinctive of our times: throughout the ages, humans have used tools that grow ever more advanced, modifying inventions of earlier generations or other communities of tool makers. Yet there seems to be something uniquely human, if not uniquely twenty-first century human, about this: no other animal species appears to have technologies or processes of innovation and diffusion that match ours.

It is difficult to deny the intuitive appeal of the bold claims made in the previous paragraph, although it might be easy to think of complications and exceptions. All of the claims also feature prominently in past and ongoing research programs on human evolution. Here, we are concerned in particular with cultural-evolutionary theory, in which the *cumulative* character of human culture provides one ground for applying Darwin’s idea of descent with modification^1^; and with approaches that regard cumulative culture or so-called ‘ratchet’ effects as uniquely human.^2^ More particularly, we focus on one standard way in which this cumulative character has been presented, namely in terms of the *complexity* of cultural traits, where technologies are taken as primary examples of such complex traits.^3^ Our leading question is whether, irrespective of intuitive appeal, the empirical evidence is sufficiently strong to support or undermine this technological complexity thesis, which holds that cumulative technological complexity is a distinctive characteristic of human cultural evolution.

This question resembles a famous predecessor. In 1991, the biologist–philosopher Daniel McShea published a now classic paper (McShea 1991, this journal), titled “Complexity and evolution: what everybody knows?”, in which he criticized the consensus among evolutionists that (morphological) complexity increases in biological evolution. Reviewing the little empirical evidence available, McShea at that time reached the skeptical conclusion that the matter was far from decided; not enough credible evidence existed to make an empirical case either for or against the increasing complexity thesis.^4^ For example, in many cases McShea found that the supposedly supportive evidence didn’t come from random samples, but was deliberately chosen in order to make a case.

As to what sustained the consensus despite the lack of decisive evidence, McShea (with Ruse 1988) conjectured that evolutionists might read into evolution the progress that seems evident in technology and science. Yet, as McShea conceded, perhaps received opinion on technology suffers from myopia just as much as received opinion on biology. Perhaps we are, just like the evolutionists McShea discussed, biased towards certain types of data, such as contemporary Western technologies, for which the technological complexity thesis seems self-evident.

In this paper, we show that the empirical case for the technological complexity thesis is surprisingly weak. Moreover, we indicate the implications this finding has on future theoretical and empirical work in the abovementioned research traditions that have been significantly shaped by this thesis.

We start by showing in more detail how the technological complexity thesis has been expressed in work in cultural-evolutionary theory (Sect. 2). In particular, we argue that different versions of the thesis have been presented as stylized facts or phenomena which cultural-evolutionary accounts or models are supposedly able to account for.

Then, in Sect. 3, we clarify what an adequate test of the technological complexity thesis should look like. Preferably, following McShea (1991), such a test would be based on a representative sample of established lines of descent that contain information on complexity (in any of the senses that have been proposed). We explain the difficulties in applying these adequacy criteria to technological evolution, but also point out reasoned strategies to overcome them.

Section 4 performs the test, relying on the few datasets that fit our purposes. We find that the data do not unequivocally point to complexity increase or decrease and that, even if they do, they do so only for very specific, and usually different, conceptualizations of complexity. Furthermore, the data fail to form a representative sample. In sum, the currently available evidence neither substantiates nor undermines the idea of a general trend towards higher complexity in technological evolution.

In Sect. 5, we draw out the implications for research in cultural-evolutionary theory, more specifically, the body of literature discussed in Sect. 2. We observe that comparative studies, in light of Sects. 3 and 4, fail to establish the accumulation of technological complexity to be uniquely human; and we highlight how future empirical work might address this issue. Furthermore, we explain that, in light of Sect. 4, it is uncertain whether cultural-evolutionary models devised to provide explanations for the evolution of complex culture target real phenomena. And, again, we point out strategies modelers might pursue to accommodate this worry. Also, we suggest that it might be worthwhile to operationalize cumulative culture in terms other than complexity, e.g., in terms of diversity, adaptivity or efficiency.

In the concluding section (Sect. 6), we discuss the relation of our empirical findings to theoretical work in biology, work that has suggested that complexity will generally increase in biological evolution (Fisher 1986; McShea and Brandon 2010). We argue that this work is compatible with finding decisive evidence against the *technological* complexity thesis. Additionally, we critically assess the ideas, also prominent among cultural evolutionists, that humans are uniquely capable of producing technologies which no single individual could invent alone and are uniquely capable of developing complex technologies. We find that both ideas are underdetermined by the available evidence.

## Technological complexity and cultural evolution

In this section, we take two steps that are preliminary to analyzing the evidence for the technological complexity thesis. Firstly, we review how advocates of cultural-evolutionary theory have endorsed or alluded to versions of the technological complexity thesis in programmatic statements and more specific (modelling) efforts. Secondly, and relatedly, we disambiguate the thesis as it features in this body of work: we distinguish different versions that require different types or measures of evidential support and that also differ in their salience for cultural-evolutionary theory. We find that, where McShea (1991) distinguished weak and strong versions of the biological complexity thesis, endorsements of minimally five versions of the technological complexity thesis may be found in the work of cultural evolutionists.

Cultural-evolutionary theory and variants such as gene-culture co-evolutionary theory and dual-inheritance theory claim that human culture is an adaptation that is pivotal in the remarkable evolutionary success of our species. Cultural traits are regarded as transmitted between generations through imitation and social learning, constituting an inheritance system that is interrelated with, but in principle independent from genetic inheritance. Here, cultural traits are understood broadly, as any information that is capable of being expressed in behavior and of being transmitted through social learning; it includes languages, legal systems, religions, artistic expressions, artisanal skills, and technologies. Much of the work within cultural-evolutionary theory consists of modelling the population-level effects of various known or supposed features of human cultural transmission—such as our propensity to imitate frequently observed traits (conformity bias) or to mimic perceived success (success bias). Such population-dynamic models of cultural evolution should, for instance, underpin the adaptiveness of specific learning strategies; explain why humans are unique among biological species in the depth and scope of social learning; or explain various ‘stylized facts’ regarding human evolution.

Technologies feature prominently in such stylized facts. We shall go on to distinguish several claims made in cultural-evolutionary theory, which may be understood as subtly different ways of developing a shared narrative or framework concerning human evolution and the role of technology. This narrative is that humans are unique among biological species in possessing a repository of traits which have gradually accumulated over many generations of human learners, and which are adaptive or functional, i.e., which facilitate or enable survival in a wide variety of specific environments. Technologies are a, or perhaps the, prime example of these products of human cultural evolution. To underline the importance of intergenerational cultural transmission, most versions of the narrative stress that technologies can not be the result of individual learning, because no single human designer, or single generation of human designers, has the capacity to invent them ‘from scratch’. Typically, the intuition that technologies have progressed beyond individual invention is captured by describing them as ‘complex’. Thus, the narrative assumes, or makes plausible, that there is a process of “gradual cultural evolution of complex, adaptive technologies” (Boyd et al. 2013: 120), that this process and its products are supposedly unique for humans and casts them as a central explanandum in cultural-evolutionary theory, or as a target phenomenon for cultural-evolutionary models.

This basic narrative is compatible with a variety of more specific claims regarding human cultural evolution and complex technologies. Here, we distinguish five versions of what we call the ‘technological complexity thesis’. Although some are more explicitly endorsed in the literature than others, each version may be illustrated with one or more quotes or paraphrases. These also illustrate that, in outlining the phenomenon, technology is predominantly described as ‘complex’, and only occasionally—often in conjunction—in other terms such as ‘diverse’, ‘efficient’ or ‘adaptive’. The five versions are:

- ‘Presence’: human cultural evolution has resulted in complex technologies.^5^
- ‘Threshold’: human cultural evolution has resulted in technologies that are so complex that they cannot be the result of individual learning.^6^
- ‘Overall increase’: human cultural evolution gives rise to, globally, ever more complex technologies—although there might be occasional, local decreases in complexity.^7^
- ‘Monotone increase’: in each successive generation, human cultural evolution results in more complex technologies.^8^
- ‘Numerically tractable increase’: human cultural evolution results in technologies that increase in complexity over successive generations in some numerically tractable pattern, e.g., linearly, or exponentially.^9^

This multiplicity of versions prompts the question which is the most salient in the context of the explanatory project outlined above. For this, we should note firstly that not each of these versions is endorsed as identifying a *unique* feature of human evolution; tellingly, the illustrative quote for thesis (a) stresses the commonality with nonhuman species, whereas some of those for theses (c) and (d) claim humaniqueness. Furthermore, it is readily apparent that theses (d) and (e) are stronger versions of, i.e., logically entail, thesis (c). Thesis (e) may be regarded as, in turn, stronger than thesis (d), since the latter does not involve any claim regarding numerically tractable patterns in complexity increase. However, many actual endorsements of thesis (e) are explicitly limited to *some* technologies, and come with the acknowledgement that, locally, the complexity of some—or, in rare cases, even all—technologies may decrease. Thus, we take thesis (d) as a qualitatively stronger version of thesis (c), and thesis (e) as a quantitatively stronger version. Similarly, the relation between theses (b) and (c) is more intricate than it might appear. At first glance, claiming the former may seem less demanding than claiming that cultural evolution more or less continuously increases technological complexity. However, whereas thesis (c) can in principle be substantiated by choosing an appropriate measure of complexity, thesis (b) requires a clear specification of some threshold value of whatever measure of complexity is chosen to substantiate thesis (c).^10^

Given these relations between the five versions and their salience for cultural-evolutionary theory’s claims regarding humaniqueness, a test of the technological complexity thesis can reasonably start by inspecting how the ‘overall increase’ thesis (c) may be substantiated (Sects. 3 and 4), before turning to the support for one of the other theses (Sect. 6).

To substantiate any version of the technological complexity thesis, several strategies are in principle available to cultural evolutionists. One is to draw on analogies between biology and culture in order to make credible a technological counterpart of a biological complexity thesis (more on this in Sect. 6). Another is to offer theoretical, e.g., model-based arguments. Perhaps surprisingly, given the frequent use of analogical arguments in support of the application of Darwinian principles and the strong orientation towards modelling, neither of these argumentative strategies is used by cultural evolutionists to substantiate any version of the complexity thesis as an explanandum. Rather, the thesis is presented as a stylized fact on the basis of a few salient examples, such as smartphones and space shuttles.

Now, it may well be the case that there is a strong empirical basis for the technological complexity thesis, which undergirds its intuitive appeal and from which a wide variety of illustrative examples could be drawn. Our question in the next two sections is: is there sufficient and appropriate empirical evidence to accept—or, alternatively, to reject—the ‘overall increase’ version of the technological complexity thesis?

## Adequacy criteria for a test of the technological complexity thesis

According to version (c) of the technological complexity thesis, complexity increases globally or on average, that is, in some lineages and more (often) than not. Accordingly, following McShea (1991), an adequate test of the thesis is one that is based on a representative sample of established lines of descent for which the complexity of ancestors and descendants has been or can be measured.

In order to apply these adequacy criteria to technology, they need to be made more precise. To start, what would count as an acceptable measure of technological complexity? In many presentations, cultural evolutionists have understood technologies straightforwardly as functional artifacts, such as kayaks, bows, and dogsleds (Boyd et al. 2013, p. 122). Other versions include or focus on bodies of knowledge (e.g., Mesoudi 2011b) rather than material items; and still others refer more or less consistently to behavioral patterns (e.g., Dean et al. 2014). This reflects a multiplicity of meanings that is widely acknowledged in the philosophy of technology (see, e.g., Mitcham 1978): ‘technology’ may refer to collections of artifacts, to bodies of knowledge, or to goal-directed practices, where none of these meanings holds obvious conceptual priority over the others. This multiplicity also surfaces in the various attempts to define technological complexity. As can be seen in Table 1, some definitions pertain to technological artifacts (definitions [1–2]), some to technological behavior (definitions [3–6]), others can be applied to both artifacts and behavior (definitions [7–9]). At a first pass, any sensible operationalization of the items on the list might be considered adequate. Yet, one might also insist that some measures are better than others. For instance, it has been claimed that humans’ unique capacity for cumulative cultural evolution can be attributed to imitation, i.e., faithful replication of the actual behavior of a mentor (e.g., Tomasello and Call 1997; Tomasello 1999; Boyd and Richerson 1996; Richerson and Boyd 2008; Mesoudi et al. 2013), rather than to emulation, i.e., re-invention of behaviors as a response to being exposed to the end products (e.g., artifacts) of others’ behaviors. If this is right—we take no stand on this issue here—and if cumulative culture refers to the accumulation of technological complexity (Mesoudi 2011a, b; Boyd et al. 2013; Kempe et al. 2014; Dean et al. 2014), the most natural definition of complexity would thus be one that pertains to technological behavior (definitions [3–6]) rather than to technological artifacts (definitions [1–2]).

**Table 1:** Definitions of technological complexity

Nr.	Definition of technological complexity	Applies to	References
[1]	Complexity expressed as the number of techno-units an artifact consists of; techno-units are the different kinds of parts in a tool	Artifacts	Oswalt (1973, 1976)
[2]	Complexity expressed as the number of tools in a toolkit	Artifact sets	Oswalt (1973, 1976)
[3]	Complexity expressed as transmission inaccuracy, i.e., the inaccuracy of learning a trait from a mentor; complex skills are those that are hard to learn, and thus have high transmission inaccuracy	Behavior	Henrich (2004) and Powell et al. (2009)
[4]	Complexity expressed as skillfulness	Behavior	Mesoudi (2011b)
[5]	Complexity expressed as the presence of multiple sets of (inexact) means-end connections	Behavior	March and Simon (1958)
[6]	Complexity expressed as the presence of interrelated, conflicting subtasks	Behavior	Campbell (1984)
[7]	Complexity expressed by the density of interactions between a system’s parts	Undefined; can be applied to artifacts and behavior	Simon (1962)
[8]	Complexity expressed as a function of the number of parts, the degree of differentiation or specialization of these parts, and their integration	Undefined; can be applied to artifacts and behavior	Service (1962)
[9]	Kolmogorov complexity of an object (e.g., a use plan) expressed as the length of the shortest description of that object	Undefined; can be applied to artifacts and behavior	Kolmogorov (1974)

One might think that the latter type of complexity is a reliable proxy for the former type. But this is far from established. Artifacts are multiply realizable. For example, prehistoric projectile points were made from stone or from osseous materials, such as bone, antler and ivory. Osseous points seem at least as complex as, if not more complex than, stone points: they have the same number of techno-units (definition [1]), also when hafted; when hafted, the densities of interactions between components plausibly are identical (artifactual definition [7]). However, since osseous materials are less subject to breakage, points made of caribou antler, are probably easier to produce than stone tools (Guthrie 1983) (behavioral definitions [3], [4] or [5]). More specifically, they can be produced by gradually scraping the raw material into the requisite form, and thus do not depend on preparatory work that is as extensive and risky as the work required for producing stone points.

In a similar vein, one cannot read off from an artifact the complexity of its usage—indeed, another type of technological behavior that is subject to imitation. For instance, because automatic gearboxes contain more components (definition [1]) and perhaps denser interactions between parts (definition [7]), they would seem more complex than manual gearboxes. But the reverse holds when we consider their use. The series of actions involved in operating a manual gearbox includes one component more than the series of actions involved in operating an automatic transmission (definition [8]), i.e., gear-shifting, and that component interacts with at least one other component in the series (definition [7]), i.e., steering the wheel.

Measures of complexity might even diverge within their own class (i.e., artifactual or behavioral). Complexity of production is not a reliable indicator of complexity of usage, as illustrated by the gearbox example. Or, to take an example from archaeology, unbarbed spears made out of one piece of wood—as those found at Schöningen (Thieme 1997)—are relatively easy to produce, but it requires an awful amount of skill and knowledge about the prey and the environment to put these one-component tools to effective use. Finally, obviously, there is no reason to expect artifactual definitions [1] and [2] to be correlated. The number of techno-units of a hafted spear tells us little about the number of other tools within the spear user’s toolkit.

Such possible divergence between measures of complexity is also relevant to specifying what would count as a representative sample for testing the complexity thesis. Version (c) of the complexity thesis comes in at least as many variants as the number of definitions in Table 1. Said divergence now tells us that for a sample to be representative it ought to match the variant under consideration.

What else would make for a representative sample? Since the accumulation of complexity is supposed to be uniquely human, the sample must preferably be drawn from the population of *all* past and present human technologies. Supposing we have agreed on a measure of complexity, we also must get a sense of whether that population contains subpopulations or strata, each of which would need to be sampled independently for the sample to be representative. Biological taxa usually serve the purpose of stratification in biology, but unfortunately no such established strata are available for technology. So whereas we might draw a subsample from all the large branches of the tree of life, we lack a tree of technology, or any other fully comprehensive classification with non-overlapping classes, allowing us to do the same for technological evolution. Consequently, we will need to perform a fully random sample, or else define strata which we, on theoretical grounds, suspect to be homogeneous in the relevant respects. Regarding such stratification, the level of technological complexity among small-scale societies has been claimed to depend on a variety of factors: environment, resource availability, subsistence, sedentism, linear settlement, technology, storage, population, exchange, conflict, competition, social organization, territoriality, style, labor organization, craft specialization, inequality, and status differentiation (Price and Brown 1985). These factors thus might guide us in subsample selection. Ideally, our sample would include a decent amount of variation with respect to these variables. If, as will turn out later, this proves unfeasible, the next best option is to seek and sample the extremes. One might for instance sample hunter-gatherer and WEIRD (Western, Educated, Industrialized, Rich and Democratic; Henrich et al. 2010) societies, as they, even taking into account internal variability, might be taken to differ substantially in a substantial number of respects, such as environment, resource availability, and sedentism. Alternatively, one might contrast samples pertaining to selectively neutral traits with samples pertaining to traits that are under intense selective pressure; functional considerations might be thought to constrain trends in complexity in the latter but not in the former.

**Fig. 1:**
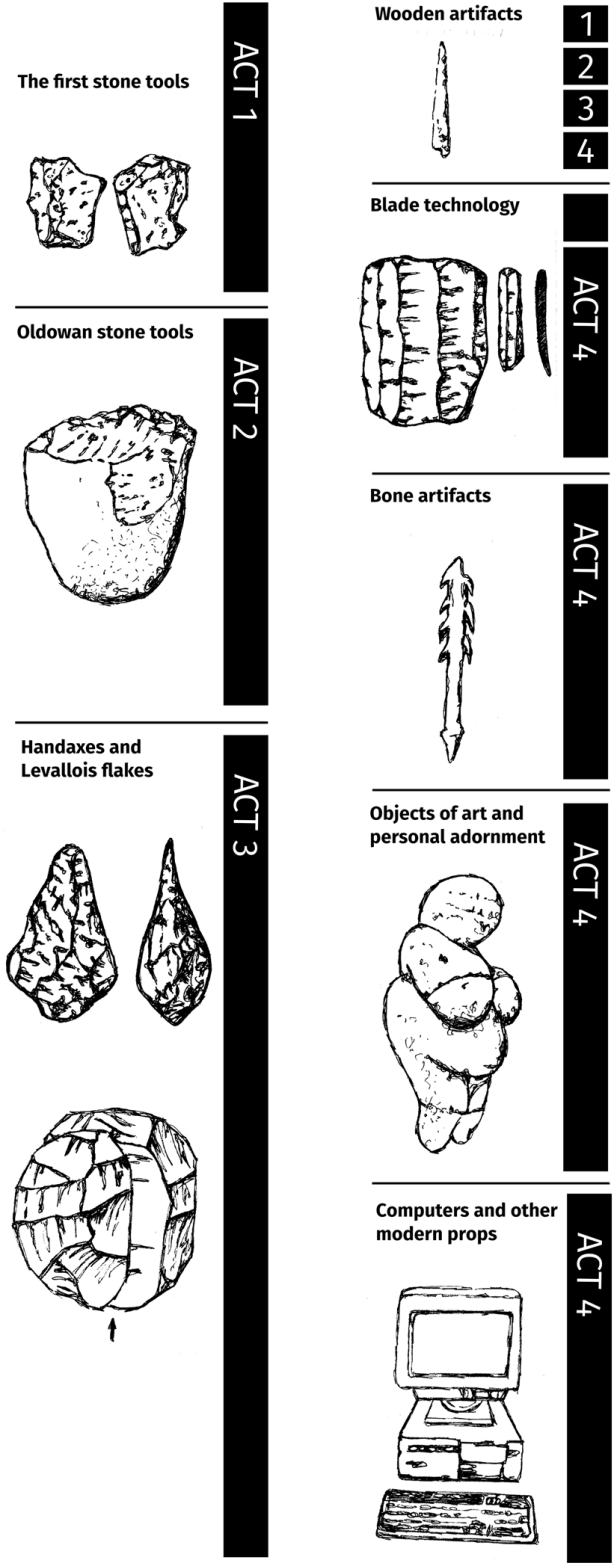
Mithen’s reconstruction of human technological evolution (redrawn from Mithen 1996)

Although such stratifications may avoid one of the problems which the lack of a well-established phylogeny of technologies gives rise to, it does not help us to address a second, thornier issue. The requirement to start from well-established lines of descent implies that one must consider, where possible, the developments in all, or at least an appropriate subset, of the lineages branching off from a given ancestor. To appreciate the importance of this point, consider Fig. 1, which is Steven Mithen’s (1996) fairly standard reconstruction of technological development over the history of our species. It starts with the first flaked stones documented by the archaeological record, dating to 2–3 million years ago, and continues until the emergence of the present-day computer. Now even if we were to agree with Mithen that the sequence evidences increasing complexity, and even if we assume that it represents actual ancestor-descendent relations (which evidently is contentious), the sequence does not establish the complexity thesis. For it includes only the tools that are supposedly diagnostic for the acts in what Mithen calls the drama of our past. So the Oldowan (2–1.5 Ma) (Act 2) is taken to be typified by the sharp-edged stones produced by striking one cobble (the core) with another (the hammerstone), as well as the remaining cores, which can be variously used as a chopper or scraper or something else. The emblematic feature of the early Acheulean techno-complex (ca. 1.6–0.5 Ma) (Act 3) is considered to be the bifacial handaxe, produced by more obviously shaping a core by means of both stone and soft hammers, whereas the late Acheulean (0.5–0.25 Ma) is typified by the emergence of Levallois tools, i.e., stone points the size and form of which is predetermined by careful preparation of the core. Mithen’s reconstruction thus ignores the diversity of tools within each act. For example, it ignores the fact that flaked tools continue to be abundantly present in the Acheulean and in fact the whole of prehistory, much later, e.g., among contemporary Aboriginals (Holdaway and Douglass 2011) and, until the 1960s, in the Mediterranean (Karimali 2005); it ignores other marked continuities, such as the persistent use of bone implements to work animal hides (Soressi et al. 2013) and unbarbed spears made out of one piece of wood—as at Schöningen (Thieme 1997) and among recent populations, for example in New Guinea and Australia (McCarthy 1957)—from the Pleistocene until present times; it ignores the fact that later stone implements, such as some of the handstones used during the Neolithic for grinding cereals, have a production process comprising only one step (i.e., seeking a suitable piece of rock; *ibid*.), which makes them, per definition [8], simpler than Oldowan tools; it ignores the fact that, as already hinted at, the use of osseous materials might have actually simplified production processes; and so forth. Obviously, these examples are insufficient to undermine the complexity thesis. Yet they do illustrate that uni-linear reconstructions fail to record developments which might shift the evidential balance, and thus which any proper test would like to include.

One might worry that technological evolution is highly reticulate, and therefore that the demand to start from established lines of descent is too stringent. Indeed, our ability to produce unambiguous rooted networks is currently limited (Morrison 2011). So perhaps we should acknowledge that, currently, we often lack the means to reliably infer technological ancestor-descendent relationships. This acknowledged, the next best option is to draw diachronic samples from a domain that preferably is wider in range than that of uni-linear reconstructions. The underlying assumption would be that, if the complexity thesis is true, one should observe an increase in complexity irrespective of the precise evolutionary relations between the diachronic samples.

## Testing the technological complexity thesis

To our knowledge, and for the moment ignoring the question of representativeness, no dataset meets the adequacy criteria discussed above. Recent work on cultural evolution has produced a number of rooted phylogenetic trees, e.g., relating to prehistoric projectile points (O’Brien et al. 2001), musical instruments (Tëmkin and Eldredge 2007), tribal textiles (Tehrani and Collard 2009), cutlery (Riede 2009), bicycles (Lake and Venti 2009) and canoes and houses (Jordan 2014). Yet none of these reconstructions contains direct information on complexity, nor is it obvious how one would infer such information. Hence, even if one grants that they accurately represent evolutionary relationships, they do not provide an appropriate test of the complexity thesis. The same holds for uni-linear sequences developed for ship rudders (Mott 1997), the cotton gin, steam engine and barbed wire (Basalla 1988), pencils (Petroski 1989), and paperclips and zippers (Petroski 1992). Some recent experimental studies provide evidence for the effect of population size on maintaining a simple or complex technology (Derex et al. 2013), where producing the complex technology requires more steps (definition [8]), but do not concern increasing complexity.

Uni-linear sequences that *do* contain information on complexity include sequences developed for early stone tools and for software packages.

**Fig. 2:**
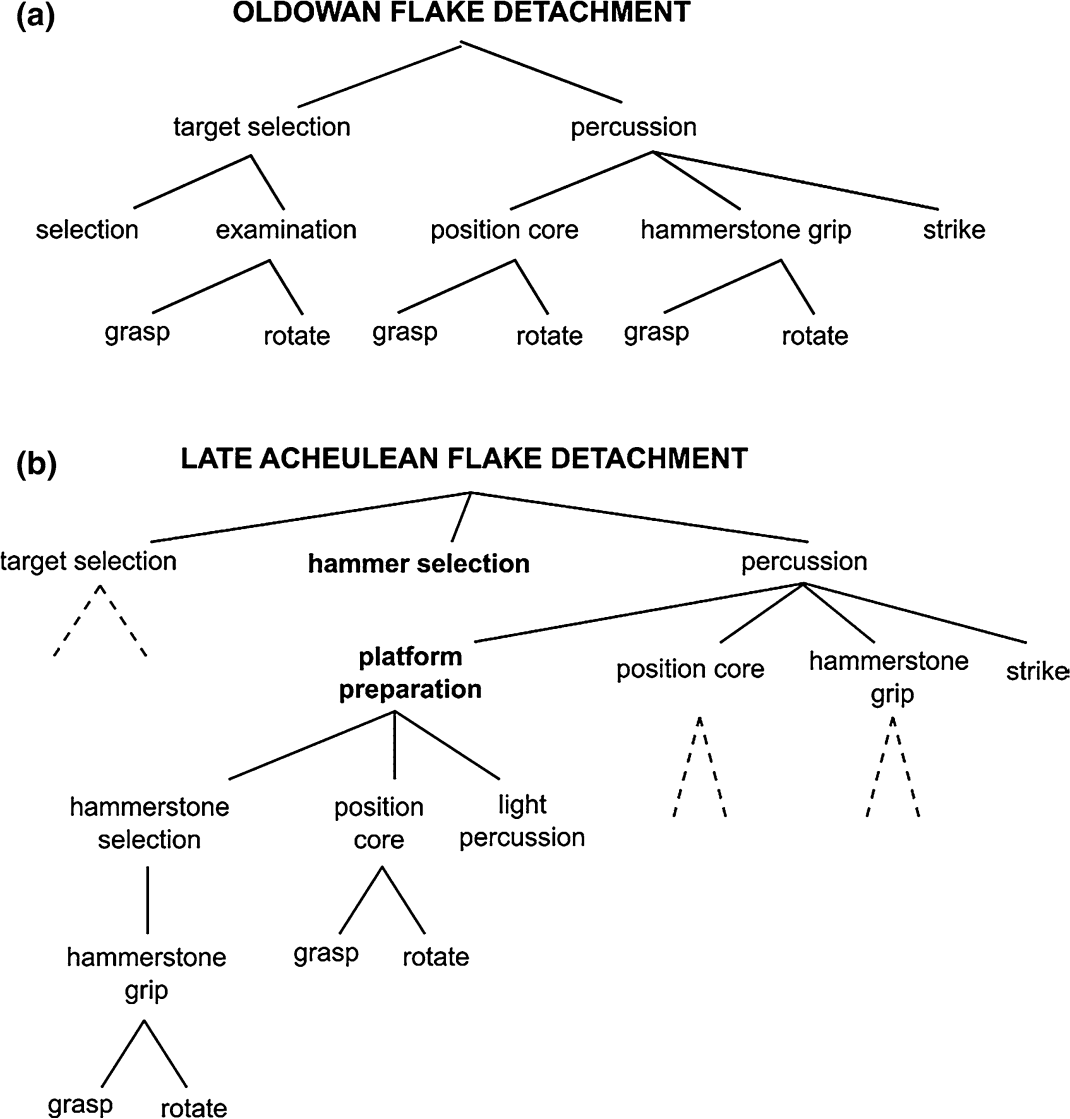
Action hierarchies for **a** Oldowan flake detachment; **b** Late Acheulean flack detachment (redrawn detail from Stout 2011). Lines connect subordinate levels with the superordinate element they instantiate. In **b**, dashed lines indicate action chunks which are identical to those defined in **a**

Regarding the former, Perreault et al. (2013) calculated the complexity of some of the diagnostic stone tools from assemblages from the Lower to Upper Paleolithic, by counting the procedural units involved in tool manufacture (definition [8]). The authors defined a procedural unit as a manufacturing step that makes a distinct contribution to the finished form of a technology (e.g., heat treatment, decortification, hard hammer percussion). Perreault et al. found that the average number of procedural units increased over time. In another study, Stout developed action *hierarchies* for various forms of stone tool production, including the production of Oldowan and Early Acheulean flakes, and Early and Late Acheulean handaxes. Consider Fig. 2 (adapted from Stout 2011), which presents action hierarchies for Oldowan and Late Acheulean flake detachment. The latter is more complex than the former for two reasons (Querbes et al. 2014). First, it has more constitutive elements (definition [8]). Second, these elements are organized in a hierarchical structure that comprises more nested levels: the addition of platform preparation to the superordinate goal of percussion in Late Acheulean flake detachment introduces four nested levels, so that the method contains six nested levels in total (versus four in Oldowan flake detachment). The success of the superordinate level (i.e., percussion) is thus contingent on four elements (rather than three, as in Oldowan production), namely position core, hammerstone grip, strike *plus* platform preparation, and the success of the latter is itself contingent on the interplay of a whole set of lower-level actions. Given this nestedness, Late Acheulean flake detachment is more complex in that it exhibits a higher density of interactions between individual actions (definition [7]); even a very small error introduced during transmission in one element may have profound repercussions on the performance of other elements, and thus on success overall. In sum, Perreault et al.’s uni-linear sequence provides support for the complexity thesis in one of its variants (i.e., corresponding to definition [8]), whereas Stout’s sequence provides support for two of the thesis’ variants (i.e., corresponding to definitions [8] and [7]).^11^

In computer science, complexity is considered to be a vice rather than a virtue; the more complex a software package, the more likely it contains a bug and the higher its maintenance costs (Sessions 2008). Hence, computer scientists have spent quite some effort on understanding the sense in which complexity might increase in software evolution. Empirical studies seem to agree that software generally grows over time (see the review in Neamtiu et al. 2013). This trend is attested by increases both in the numbers of lines a software package comprises (definition [1] and [9]) and in the number of modules contained in a software package (definition [1]). With regard to measures which computer scientists take to be genuine indicators of complexity, the picture is mixed. There is some evidence that interface complexity, which is a function of the number of input parameters to a function and the number of output states from the function (plausibly, definition [1]), might be decreasing (Bhattacharya and Neamtiu 2011). Common coupling, which is a metric for the strength of relationships between functional units (such as modules), might increase in absolute terms, but not when normalized over the total number of possible couplings (Neamtiu et al. 2013). Note that since according to definition [7] it is the *density* rather than the sheer number of interactions that counts for something to be complex, it is the trends in *normalized* common coupling that provides a suitable test of the complexity thesis (i.e., understood in terms of definition [7]). The available evidence for so-called cyclomatic complexity also is mixed. Cyclomatic complexity is measured by the number of independent paths through a program’s code. For instance, if a source code contains no conditionals, such as if-else statements, there is only one path from start to end, and the code’s cyclomatic complexity would be one. If it contained one such conditional, there are two possible paths through the code, and cyclomatic complexity would be 2. Cyclomatic complexity thus resembles closely the multiplicity of means-end connections that is central to definition [6]. It has been found to increase (Neamtiu et al. 2013) and decrease (Bhattacharya and Neamtiu 2011) in absolute terms; when normalized, cyclomatic complexity does not appear to increase (Neamtiu et al. 2013).

**Fig. 3:**
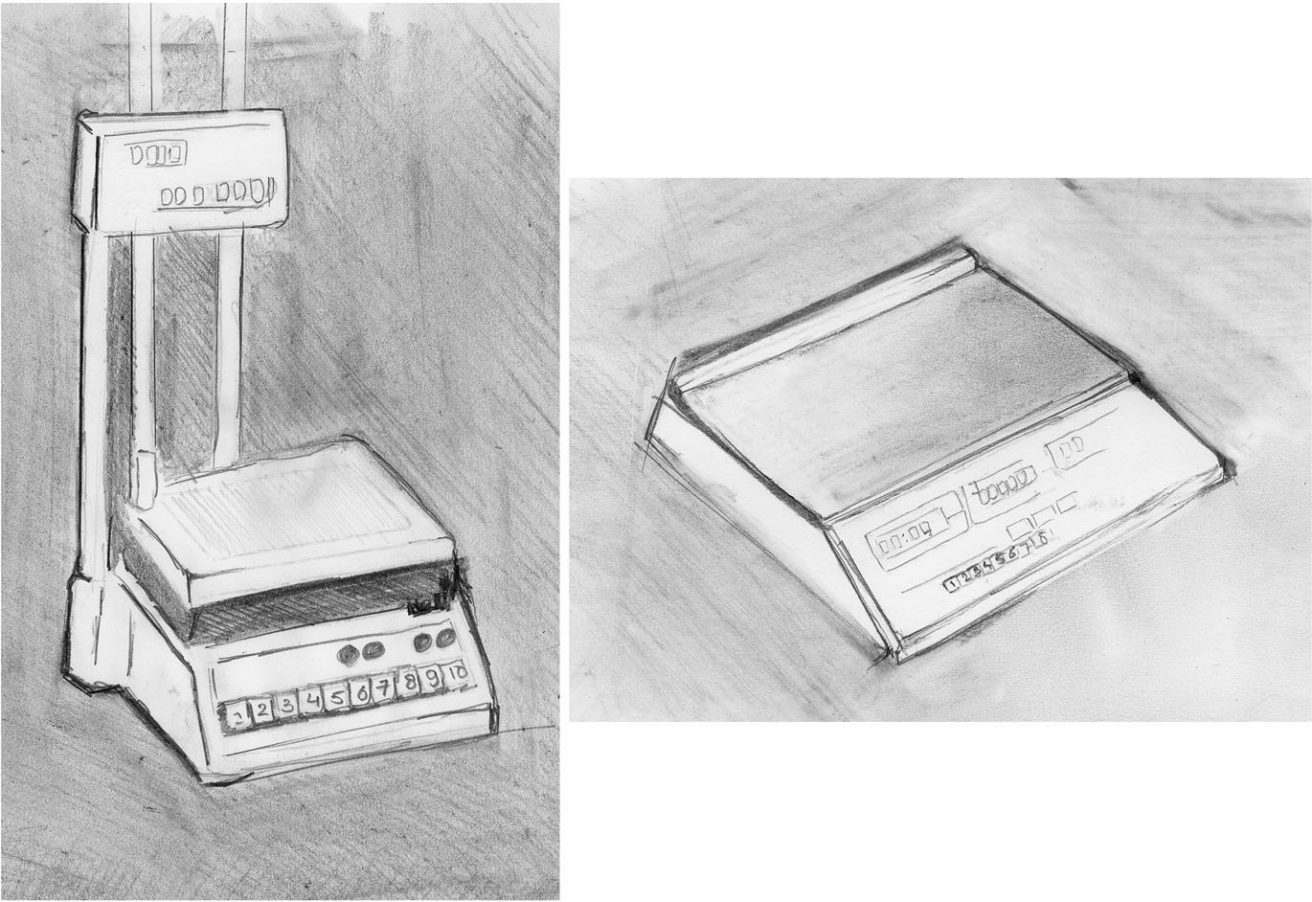
The Avery 1770 (left) and the Avery A600 (right), redesigned for ease of manufacture (redrawn from Corbett et al. 1991)

One possible reason for the lack of unambiguous evidence for complexity increase might be that software developers are sensitive to the risks such increases pose (i.e., more bugs, higher maintenance costs), and thus at least attempt to ‘design for simplicity’. A similar strategy is pursued in other industries, usually under the banner of ‘design for manufacture’. Here, the idea is to design products in such a way that their production is easy and cheap. Consider a case presented by Corbett et al. (1991; see also Vaesen 2011). It concerns the design of simple scales, used in the retail business. In 1983, W&T Avery Ltd. introduced the Avery 1770 (see Fig. 3, left), a scale that soon could be seen in shops throughout the UK. Although one expected world market volumes to grow, the company also expected competition to increase. Therefore it immediately started to redesign the 1770, explicitly bearing ease of manufacture in mind. The effort led in 1986 to a new scale, the A600 (see Fig. 3, right). The scales were functionally equivalent, but in the A600 the number of components was reduced substantially (see Fig. 3) (definition [1]). This, in turn, reduced assembly time and created benefits relating to production control, timely delivery, quality, investments, the supply chain and inventory of parts and final products. In a similar vein, in order to reduce production costs engineers might ‘design for modularity’, i.e., produce designs which limit or cluster interactions between components (definition [7]) (Baldwin and Clark 2000). In sum, engineers have certainly understood that complexity of production processes and finished products comes at a price and, accordingly, have come up with various strategies for simplification.

Another class of studies does not track ancestor-descendent relationships, but compares the complexity of elements within two or more diachronic samples. We know of three such studies. In a first study, Moore et al. (2009) examined the stone tool assemblages found in five stratigraphic layers (spanning some 95 k years, viz. ∼100 to ∼5 kya) from a cave in West Flores. Moore et al. drew a large sample of artifacts from each stratigraphic layer (11,667 artifacts in total) and counted the type and number of percussion blows needed to produce the artifacts. The authors observed that the median number of blows (definition [8]) across the assemblages remained constant. This is not to say that during the Holocene, as evidenced by the latest of the assemblages, no changes occurred: there was a higher reliance on unifacial flaking, the appearance of edge-glossed flakes, a change in raw material selection and more frequent fire-induced damage to stone artifacts. However, Moore et al. did not argue, nor provided any reasons to assume, that these changes mark increasingly complex modes of production: unifacial flaking and changes in raw material selection are presented as simply different modes of production; edge-glosses are plausibly due to characteristics of the material (viz. chert) and to usage; and there is no evidence that fire-induced damage is the result of deliberate heat treatment. To be sure, elsewhere on Flores Holocene deposits often contain edge-ground, rectangular-sectioned adzes, artifacts which Moore et al. claim (but do not demonstrate) to be more complex. So future study might very well reveal that stratigraphic layers containing such adzes are more complex (as measured by the median average of blows needed to produce the artifacts within the stratigraphy) than Pleistocene deposits. At present, however, no such evidence is available.

In a second study, Lindenfors et al. (2015) examined the development of European cooking recipes from medieval to modern times, and found evidence for increasing complexity. Over time, recipes documented by cookery books for instance tended to involve more steps (definition [8]) and a wider variety of techniques (possibly, definition [2] or [5]), and dishes tended to contain more ingredients (definition [1]).

Finally, third, Strumsky et al. (2012) conclude, based on patent claims from the period 1835–2005, that the patent record documents growing complexity of technological inventions. This conclusion is inferred from the observation that patents, over time and on average, tend to be assigned to a higher number of patent codes, and this might be taken to imply that patented technologies comprise a higher number of constitutive elements (definition [1]). To illustrate the relationship between the number of codes and the number of constitutive elements of a patented invention, consider one of the examples provided by Strumsky et al. Patent number 6,823,725 (filed on 12 December 2000 and granted on 30 November 2004) concerns a ‘Linear distance sensor and the use thereof as actuator for motor vehicles’. This patent is categorized by three codes, namely 73/114.01, 324/207.13 and 324/207.24. Code 73/114.01 implies that the invention involves a process or apparatus to perform a specific test on combustion engines; code 324/207.13 indicates that it involves a process or apparatus for measuring changes in a magnetic field; and code 324/207.24 specifies that it involves a process or apparatus for measuring motion in a straight line. This example, as well as the other examples in the study by Strumsky et al. (see their Appendix I), thus suggest that patent codes might be a reasonable proxy for the number of elements a patented invention comprises.

Table 2 summarizes our review. Recall that in order to assess the support for the complexity thesis, we must assess the support for specific variants of version (c) of the complexity thesis. We now observe that, for most of these variants, we have either no evidence (variants [3] and [4]), evidence pertaining to only one technological domain (variants [2], [5], [6] and [9]), or evidence pertaining to two technological domains ([7], [8]), one of which is ambiguous in its support. The broadest sample is available for variant [1]. But also here the sample includes one domain (out of three) with ambiguous results. Surely, finding one ambiguous result in a representative sample would not invalidate a thesis according to which technological complexity can be expected to increase *on average*. Yet, the fact that the sample for variant [1] is not representative—it is a WEIRD sample, which might furthermore inadequately represent some tendencies within WEIRD technologies, such as a trend towards design for simplicity, manufacture or modularity—does allow us to conclude that what McShea observed in biological evolution also applies to technology: insufficient credible evidence exists to make a forceful empirical case either for or against the complexity thesis.

**Table 2:** Overview of results (+, results supporting the complexity thesis; ±, ambiguous results)

Def. nr.	Technological domain			
	Stone tools	Software	Cooking recipes	Patents
[1]		±	+	+
[2]			+	
[3]				
[4]				
[5]			+	
[6]		±		
[7]	+	±		
[8]	±		+	
[9]		+		

## Implications for research on cumulative culture

A recent paper by Dean et al. reviews for various species the evidence for cumulative culture, which the authors characterize as “the modification, over multiple transmission episodes, of cultural traits *resulting in an increase in the complexity or efficiency of those traits*” (Dean et al. 2014, p. 287, italics added). From an early study by Lehman (1947) and a study by Basalla (1988) Dean et al. infer that “human culture is clearly cumulative” (Dean et al. 2014, p. 287). For other animals (chimpanzees, capuchin monkeys, macaques, crows), the authors argue, the evidence is inconclusive.

Note that, irrespective of our previous arguments, the studies by Lehman (1947) and by Basalla (1988) fail to support the complexity thesis. Lehman (1947) counted the “outstanding contributions” in eleven academic fields (chemistry, genetics, geology, mathematics, and so forth) and plotted them over time. Unsurprisingly, the number of outstanding contributions started to rise at the onset of the scientific revolution. Basalla (1988) examined the evolution of technologies such as steam and internal combustion engines, the transistor, lighting systems, and the electric motor, and argued that all of these technologies were developed by building on previous inventions. Crucially, neither the observation of exponential growth of outstanding contributions (Lehman) nor the observation of novel artifacts arising from antecedent artifacts (Basalla) tells us that complexity increases in human cultural evolution.

Section 4 provides an additional reason for not understanding cumulative culture in terms of increased complexity. For the available evidence reviewed there appears inconclusive, perhaps as inconclusive as Dean et al.’s evidence for non-human animals. Furthermore, Sect. 3 presented an argument which casts doubt on how Dean et al. compare humans and non-human animals. Assuming that the studies by Lehman (1947) and Basalla (1988) actually did document increasing complexity, they would do so for end-products, whereas most of the evidence for non-human animals pertains to behavioral patterns (for chimpanzees: nut-cracking, ectoparasite manipulation, digging of wells; for capuchins: various social games; for macaques: stone-handling). Only the evidence Dean et al. (2014) discuss for New Caledonian crows’ leaf tools matches the type of evidence they discuss for humans. In sum, regardless of what the individual studies on humans and non-humans conclude, Dean et al.’s comparison is too uneven to establish whether and in which sense humans are unique.

Future work on the humaniqueness of cumulative culture would therefore seem to have three options. The first is to operationalize cumulation in terms other than complexity. For example, it might be less difficult to substantiate the claim that, generally, human culture develops by modifying or recombining previous inventions, or the claim that technological evolution tends towards increasing diversity (see e.g., Shea 2011). The evidence reviewed in Sect. 3 at least seems compatible with these claims, and other relevant evidence might be easier to come by.

A variant of this first option is suggested by the abovementioned characterization of cumulative culture as “an increase in the complexity *or efficiency* of those traits” (Dean et al. 2014: 287, italics added) or, relatedly, as a “gradual cultural evolution of complex, *adaptive* technologies” (Boyd et al. 2013: 120, italics added). Whereas it might be difficult to substantiate increases in adaptivity, some studies (e.g., Morris 2013 and references therein) indeed suggest that humans might have become increasingly efficient in capturing energy in the form of food, fuel and raw materials.^12^

Note that shifting the focus from complexity to diversity or efficiency (or adaptivity) is more than a terminological manoeuvre, since it entails a shift in the types of evidence one should be looking for. While counting techno-units might be relevant to substantiating the complexity thesis, it is irrelevant to testing increasing efficiency. Consider the harpoons used by contact-era Inuit to hunt seals in open water (Vaesen et al. 2016). Such harpoons typically had floats attached to them to make it more difficult for the seal to dive. Adding an extra float would increase the harpoon’s number of techno-units, but would decrease its level of energy capture (sensu Morris), because the object would be more difficult to aim. In sum, it makes good sense not to lump together concepts such as complexity and diversity and efficiency, just as it appears to have made good sense not to lump together in Sects. 3 and 4 various operationalizations of the notion complexity.

A second option for future work on humaniqueness of cumulative culture is to retain the focus on complexity, but to try and match the evidence pertaining to humans to the evidence pertaining to non-human animals. Given the fact that studies on non-human animals have predominantly focused on behavior, future work on humans should do the same—and, unlike recent transmission-chain experiments (Caldwell and Millen 2008, 2010), should do so with the aim of demonstrating increasing complexity rather than effectiveness.

A third option is the reverse of the second, i.e., trying to find evidence in non-human animals for the patterns observed in humans (see Sect. 3). We have seen, however, that these patterns are poorly documented, so that it is unclear what precisely one should be looking for in non-human animals.

The considerations presented in Sects. 2, 3 and 4 also have implications for cultural-evolutionary models devised to understand which factors might drive the evolution of complex culture. Such models differ considerably in how they construe complexity. Characterizations have been given in terms of definition [1] (e.g., Lehmann and Wakano 2013; Querbes et al. 2014), in terms of definition [3] (e.g., Henrich 2004; Powell et al. 2009; Vaesen 2012), in terms of definition [7] (Querbes et al. 2014) and in terms of definition [8] (e.g. Lewis and Laland 2012). Given the poor support for the complexity thesis (see Sect. 4), it is unsure whether any of the said models actually target real phenomena. Support for the definition assumed by the models of Lehmann and Wakano (2013) and Querbes et al. (2014) (namely definition [1]) seems strongest; yet even here we found only three supportive cases (i.e., cooking recipes, software evolution, patents), all coming from WEIRD societies.

Modellers may pursue three strategies to accommodate the concerns raised here. The first is, again, to operationalize cumulation differently (e.g., in terms of diversity, efficiency or adaptivity). The second is to try to establish, empirically, that the type of complexity increase targeted by their models is real. The third and final strategy is to perform robustness analyses, i.e., test whether models yield similar results under varying characterizations of complexity. By way of illustration, consider a study by Querbes et al. (2014). The authors assessed two models (developed by Henrich 2004; Powell et al. 2009) according to which demography is a key driver of cultural complexity. Whereas these models assumed complexity to be captured by transmission inaccuracy (definition [3]), Querbes et al.’s adaptation of the models assumed complexity to be a function of the density of interactions between functional parts (definition [7]). Querbes et al. found that, under definition [7], demography plays a much more limited role in sustaining the accumulation of complexity. Consequently, since empirical evidence is not sufficiently abundant to prefer one of the two definitions (see Sect. 4), the models do not allow us to infer that demography is a key driver of complexity. Conversely, convergence of results under a wide variety of definitions would (!) increase our confidence in the demography hypothesis.

## Discussion

The evidence for and against version (c) of the technological complexity thesis (i.e., ‘overall increase’) appears to be scarce and inconclusive. This finding is hard to reconcile with what many of us believe, often quite strongly, about progress in science and technology.

So, where does this consensus view come from? McShea (1991) offers several hypotheses regarding the origins of the consensus view in biology, and these seem to be sensible also with respect to technology. Perhaps we possess a cognitive algorithm that correctly outputs increasing complexity, but employs a conception of complexity which differs from those used in current empirical work. Perhaps items simply look more and more different from contemporary ones as we go further and further back in time; if contemporary items are assumed to be complex, less familiar items would mistakenly be judged simpler. Or perhaps we are biased towards a few spectacular cases (e.g., the Apollo mission), and accordingly get the impression of a universal, long-term trend.

To the extent that one finds plausible the analogy between biological and technological evolution, our findings are also hard to reconcile with theoretical work in biology. This work has suggested that even if complexity in every lineage follows a simple random walk (i.e., complexity decreases are as likely as complexity increases), and if there is a lower bound to complexity, the mean complexity of all lineages will rise (Fisher 1986; McShea 1991, 1994); and it has suggested that in the absence of constraints or forces, complexity will increase in a lawful manner (McShea and Brandon 2010).

There are obvious reasons to mistrust the analogy between biological and cultural evolution. McShea and Brandon (2010, p. 132), for instance, find it plausible that in cultural evolution quite a few homogenizing forces are at work. Consequently, trends in technological complexity plausibly will not conform to a law that is developed for cases in which no forces are at work. Likewise, even if Fisher’s work would theoretically establish the complexity thesis in biology, it would only apply to technological evolution if none of the following defeating conditions obtains: decreases being more likely than increases, decreases being larger in size than increases, and there not being a lower bound to complexity. Since none of these conditions can be ruled out empirically, theoretical work in biology certainly leaves open the possibility of finding conclusive evidence against the complexity thesis in technology.

What Fisher’s paper *does* show, however, is that complexity, at least theoretically, can increase even in the absence of high-fidelity transmission. Even when cultural learners *never* copy the exact behavior of their cultural parent (e.g., complexity increases and decreases have equal probabilities of 0.5), Fisher’s work predicts that, in the absence of defeating conditions, complexity will increase. Hence, from the mere fact that human culture is characterized by the accumulation of complexity one should not infer, as some authors seem to do (e.g., Tomasello and Call 1997; Tomasello 1999; Boyd and Richerson 1996; Richerson and Boyd 2008; Mesoudi et al. 2013), that such accumulation is the result of imitation, i.e., faithfully replicating actual behavior of a mentor.

An alternative for advocates of the technological complexity thesis is to abandon version (c), and focus instead on versions (a) (i.e., ‘Presence: human cultural evolution has resulted in complex technologies) and (b) (i.e., ‘Threshold’: human cultural evolution has resulted in technologies that are so complex that they cannot be the result of individual learning). As indicated above, at least one of these—version (b)—is still associated with the salient explananda regarding humaniqueness. Yet as we made clear in Sect. 2, endorsing version (b) does not reduce the need for evidential support. On the contrary, it requires a non-arbitrary specification of a threshold value—and version (a) requires clear application criteria for the labels ‘simple’ and ‘complex’. Moreover, as argued in the previous section, substantiating claims of humaniqueness would require an unbiased test of non-human cultural traits with regard to whichever application criteria or threshold value has been established. Both McShea’s hypotheses concerning the origins of the consensus view in biology and associations with the problematic distinction between ‘primitive’ and ‘advanced’ cultures give reasons for being cautious regarding version (a). Although—or perhaps precisely because—the ‘presence’ claim seems intuitively obvious, there is a real risk that the claim merely expresses perceptions of readily observable but superficial differences, or ignorance on the part of the person claiming complexity for her own cultural traits.

In this regard, operationalizing version (a) through the ‘beyond-individual-invention-from-scratch’ version (b) may look like a promising way forward. For this version, however, our discussion of Dean et al. (2014)’s claims regarding humaniqueness points out some of the complications: as obvious as (b) might seem for Jumbo Jets or smartphones, comparison between human and non-human culture needs to be on an equal (i.e., artifact vs artifact or behavior vs behavior) footing. Moreover, and in closing, the evidential basis for (b) should not be overrated. For most technologies, re-invention from scratch is practically unnecessary, but perhaps not beyond individual capacity. Not coincidentally, cultural evolutionists typically provide indirect evidence, in the form of ‘lost European explorer’ narratives (e.g., Boyd et al. 2013): historical anecdotes of expeditions of well-trained, presumably intelligent people failing in environments where indigenous people manage to survive; here, a combination of motivation, available resources and cognitive capacities was presumably insufficient to re-invent local subsistence technologies from scratch. A more rigorous search for evidence would need to establish which technologies lie outside what has recently been called the ‘zone of latent solutions’ (Tennie et al. 2016): behavioral patterns that may be triggered in sufficiently motivated individuals by social and environmental cues. Scenarios can be imagined in which this may be tested for various technologies. One is the “Island Test” proposed by Tomasello (1999): a scenario in which someone is separated at birth and raised alone on an island, but provided with sufficient motivation and raw materials to trigger and facilitate re-invention of a technology in case it is in the ‘zone of latent solutions’. As the difficulties in realizing this scenario should make clear, currently even our knowledge regarding this form of technological complexity is easily overstated.

## Footnotes

1 E.g., “Darwin famously described biological evolution as ‘descent with modification’. (…) We can similarly demonstrate the gradual accumulation of modifications in culture. (…) [S]uccessful innovations are always slight modifications of what went before, or the combination of previously separate innovations” (Mesoudi 2011a, p. 33).

2 “Human culture … has the distinctive characteristic that it accumulates modifications over time (what we call the ‘ratchet effect’)” (Tennie et al. 2009: 2405).

3 E.g., “Human culture … shows often astounding complexity, such as… technological artefacts such as computers and space shuttles that are composed of countless functionally interlinked parts” (Mesoudi 2011a, p. ix).

4 By 2010, McShea seemed to have become less skeptical, since in their book “Biology’s first law”, McShea and Brandon approvingly discuss a substantive body of recent work that might confirm the complexity thesis (McShea and Brandon 2010, p. 50).

5 E.g., “the cultural evolution of human technology is similar to the genetic evolution of complex adaptive artifacts in other species, things like bird’s nests and termite mounds” (Boyd et al. 2013, p. 120).

6 E.g., “Individuals are smart, but most of the cultural artifacts that we use … are far too complex for even the most gifted innovator to create from scratch” (Richerson and Boyd 2005, p. 53); “highly complex adaptations arise by cultural evolution even though no single innovator contributes more than a small portion of the total” (Boyd et al. 2013, p. 122); “Human cumulative culture combines high-fidelity transmission of cultural knowledge with beneficial modifications to generate a ‘ratcheting’ in technological complexity, leading to the development of traits far more complex than one individual could invent alone” (Dean et al. 2014: p. 284); “human culture [provides] knowledge accumulated across many generations that an individual alone could never produce” (Tennie et al. 2009: 2411).

7 E.g., “The tools essential for life … evolve, gradually accumulating complexity through the aggregate efforts of populations of individuals, typically over many generations” (Boyd et al. 2013, p. 120); “a characteristic feature of human cultural evolution is the seemingly limitless appearance of new and increasingly complex cultural elements” (Enquist et al. 2011); “only humans appear to possess cumulative culture, in which cultural traits increase in complexity over successive generations” (Kempe et al 2014: 29; abstract); “Of course, cultural evolution may also lead to a decrease in the efficiency, amount and complexity of culture” (Enquist and Ghirlanda 2007: 48).

8 E.g., “the modification, over multiple transmission episodes, of cultural traits resulting in an increase in the complexity or efficiency of those traits” (Dean et al. 2014: 287); “but human culture appears unique in that it is cumulative, i.e. human cultural traits increase in diversity and complexity over time” (Lewis and Laland 2012: 2171). This monotone increase is also suggested by labelling the gradual accumulation of cultural complexity the ‘ratchet effect’. E.g., “This ratcheting up in the complexity of cultural traits, frequently across multiple generations, has been proposed to be the hallmark of human culture” (Dean et al. 2014: 285).

9 E.g., “Why does human culture increase exponentially?” (Enquist et al. 2008; title); “the original model exhibits a linear increase in cultural complexity continuing to infinity” (Mesoudi 2011b: 5).

10 This does not mean that thesis (c) entails (b). In principle, technologies could have been too complex for individual invention from the dawn of humankind, so that human cultural evolution has not strictly speaking resulted in such technologies. There is a strong suggestion in cultural-evolutionary work, however, that technologies started out simple enough to be invented from scratch.

11 Some authors (Shott 2003 and references therein) have argued that stone tool reduction sequences, as the ones developed by Perreault et al. (2013) and by Stout (2011), contain an unacceptable level of arbitrariness. Such sequences would thus be inadequate for the purpose of testing the complexity thesis. We ignore this complication, however, and rather try to present the strongest case for the complexity thesis. To anticipate Sect. 4, we will find that the support for the complexity thesis is weak, even without having to invoke methodological issues of the sort raised by Shott and others.

12 We thank an anonymous reviewer for pointing this out to us. Note, also, that adaptive complexity, a notion that cultural evolutionists often allude to, in fact refers to two separate qualities (complexity and adaptivity) rather than to one. So an adaptive, complex trait is a trait that is both complex and adaptive, not one that is, conjunctively, adaptively-complex.
